# Supplementary material for: Role of marriage, motherhood, son preference on adolescent girls’ and young women’s empowerment: Evidence from a panel study in India
Source: PLoS One. 2023 Sep 28;18(9):e0292084. doi: 10.1371/journal.pone.0292084 (PMC10538655; doi:10.1371/journal.pone.0292084)
Supplement: S2 Checklist — (DOCX) [file pone.0292084.s002.docx]

STROBE Statement—checklist of items that should be included in reports of observational studies

|  | Item No. | Recommendation | Page  No. | Relevant text from manuscript |
| --- | --- | --- | --- | --- |
| **Title and abstract** | 1 | (*a*) Indicate the study’s design with a commonly used term in the title or the abstract | 2 | … prospective cohort panel dataset… |
|  |  | (*b*) Provide in the abstract an informative and balanced summary of what was done and what was found | 2 | Using two waves of data from prospective cohort panel dataset that followed unmarried (6,065 observations in each wave) and married AGYW (3,941 observations from each wave) over a three-year period from Uttar Pradesh and Bihar, we examined how marriage, childbearing, and having a son is associated with changes in AGYW's empowerment, especially considering whether AGYW marry into patrilocal households (household with in-laws) as an effect modifier. Empowerment indicators included freedom of movement or mobility, decision-making power, access to economic using Kabeer’s framework as our theoretical approach. |
| Introduction | | | |  |
| Background/rationale | 2 | Explain the scientific background and rationale for the investigation being reported | 3-5 | Life events such as marriage and childbirth may have an influence on empowerment as they shift household dynamics and change the life trajectory of women. However, prior empirical work examining the effects of marriage and childbirth on women’s empowerment has been largely cross-sectional, with empowerment being assessed as a static measure. A limitation of this approach is that it does not acknowledge empowerment as a dynamic process in which prior and present life experiences may influence empowerment later. This study addressed these gaps by using a dataset that followed a panel of unmarried and married AGYW over a three-year period. We examined how marriage, childbearing, and specifically bearing a son affects changes in AGYW's empowerment in the early part of married life in India. |
| Objectives | 3 | State specific objectives, including any prespecified hypotheses | 8-9 | Is marriage associated with a change in empowerment of AGYW? Further, does the relationship between marriage and empowerment depend on whether the household arrangement is patrilocal (newly married women co-residing with in-laws)? Is motherhood associated with a change in empowerment of married AGYW? Is having a son associated with a greater change in empowerment among married AGYW compared to having daughters only? |
| Methods | | | |  |
| Study design | 4 | Present key elements of study design early in the paper | 9 |  |
| Setting | 5 | Describe the setting, locations, and relevant dates, including periods of recruitment, exposure, follow-up, and data collection | 9-10 | Two waves of panel data were collected: Wave 1 in 2015-16 and Wave 2 in 2018-19. Wave 1 surveyed 20,594 unmarried boys and girls aged 10-19 and married girls aged 15-19 from a state-representative random sample of 300 urban and rural primary sampling units (PSUs)—villages in rural areas and census wards in urban areas. |
| Participants | 6 | (*a*) *Cohort study*—Give the eligibility criteria, and the sources and methods of selection of participants. Describe methods of follow-up  *Case-control study*—Give the eligibility criteria, and the sources and methods of case ascertainment and control selection. Give the rationale for the choice of cases and controls  *Cross-sectional study*—Give the eligibility criteria, and the sources and methods of selection of participants | 9-11 | Cohort study (longitudinal) |
|  |  | (*b*) *Cohort study*—For matched studies, give matching criteria and number of exposed and unexposed  *Case-control study*—For matched studies, give matching criteria and the number of controls per case |  |  |
| Variables | 7 | Clearly define all outcomes, exposures, predictors, potential confounders, and effect modifiers. Give diagnostic criteria, if applicable | 12-14 | Exposures: Marriage, motherhood, and mother of son  Outcomes: Freedom of movement or mobility, Decision-making power, Access to economic resources  Effect modifier: co-residence with in-laws |
| Data sources/ measurement | 8* | For each variable of interest, give sources of data and details of methods of assessment (measurement). Describe comparability of assessment methods if there is more than one group | 12-14 |  |
| Bias | 9 | Describe any efforts to address potential sources of bias | 14 | Omitted variable bias |
| Study size | 10 | Explain how the study size was arrived at | 9-11 |  |

Continued on next page

| Quantitative variables | 11 | Explain how quantitative variables were handled in the analyses. If applicable, describe which groupings were chosen and why | 11-13 |  |
| --- | --- | --- | --- | --- |
| Statistical methods | 12 | (*a*) Describe all statistical methods, including those used to control for confounding | 14-15 |  |
|  |  | (*b*) Describe any methods used to examine subgroups and interactions |  |  |
|  |  | (*c*) Explain how missing data were addressed |  |  |
|  |  | (*d*) *Cohort study*—If applicable, explain how loss to follow-up was addressed  *Case-control study*—If applicable, explain how matching of cases and controls was addressed  *Cross-sectional study*—If applicable, describe analytical methods taking account of sampling strategy | 9-10 | The re-contact rates of 79% and 82% in both these samples meet the criteria of minimum acceptable re-contact in longitudinal studies |
|  |  | (*e*) Describe any sensitivity analyses |  |  |
| Results | | | | |
| Participants | 13* | (a) Report numbers of individuals at each stage of study—eg numbers potentially eligible, examined for eligibility, confirmed eligible, included in the study, completing follow-up, and analysed | 10-11 |  |
|  |  | (b) Give reasons for non-participation at each stage | 10-11 |  |
|  |  | (c) Consider use of a flow diagram |  |  |
| Descriptive data | 14* | (a) Give characteristics of study participants (eg demographic, clinical, social) and information on exposures and potential confounders | 18 | Table 1 |
|  |  | (b) Indicate number of participants with missing data for each variable of interest |  |  |
|  |  | (c) *Cohort study*—Summarise follow-up time (eg, average and total amount) |  |  |
| Outcome data | 15* | *Cohort study*—Report numbers of outcome events or summary measures over time | 18 |  |
|  |  | *Case-control study—*Report numbers in each exposure category, or summary measures of exposure |  |  |
|  |  | *Cross-sectional study—*Report numbers of outcome events or summary measures |  |  |
| Main results | 16 | (*a*) Give unadjusted estimates and, if applicable, confounder-adjusted estimates and their precision (eg, 95% confidence interval). Make clear which confounders were adjusted for and why they were included | 19-20 | Table 2,3,4 |
|  |  | (*b*) Report category boundaries when continuous variables were categorized |  |  |
|  |  | (*c*) If relevant, consider translating estimates of relative risk into absolute risk for a meaningful time period |  |  |

Continued on next page

| Other analyses | 17 | Report other analyses done—eg analyses of subgroups and interactions, and sensitivity analyses |  |  |
| --- | --- | --- | --- | --- |
| Discussion | | | | |
| Key results | 18 | Summarise key results with reference to study objectives | 21-23 |  |
| Limitations | 19 | Discuss limitations of the study, taking into account sources of potential bias or imprecision. Discuss both direction and magnitude of any potential bias | 24 |  |
| Interpretation | 20 | Give a cautious overall interpretation of results considering objectives, limitations, multiplicity of analyses, results from similar studies, and other relevant evidence | 21-23 |  |
| Generalisability | 21 | Discuss the generalisability (external validity) of the study results | 25 |  |
| Other information | |  | | |
| Funding | 22 | Give the source of funding and the role of the funders for the present study and, if applicable, for the original study on which the present article is based | N/A |  |

*Give information separately for cases and controls in case-control studies and, if applicable, for exposed and unexposed groups in cohort and cross-sectional studies.

**Note:** An Explanation and Elaboration article discusses each checklist item and gives methodological background and published examples of transparent reporting. The STROBE checklist is best used in conjunction with this article (freely available on the Web sites of PLoS Medicine at http://www.plosmedicine.org/, Annals of Internal Medicine at http://www.annals.org/, and Epidemiology at http://www.epidem.com/). Information on the STROBE Initiative is available at www.strobe-statement.org.
