# Supplementary material for: Role of marriage, motherhood, son preference on adolescent girls’ and young women’s empowerment: Evidence from a panel study in India
Source: PLoS One. 2023 Sep 28;18(9):e0292084. doi: 10.1371/journal.pone.0292084 (PMC10538655; doi:10.1371/journal.pone.0292084)
Supplement: S1 File — (DOCX) [file pone.0292084.s003.docx]

**S1 File. Supplementary Information-Model specifications**

**Equation used to estimate association between the transition to motherhood and changes in empowerment**

To estimate the association between the transition to motherhood and changes in empowerment, we ran similar individual linear fixed-effects regressions on the sample of married AGYW for each of the domains of empowerment over time as listed below:

$$Y_{it}=\beta_{1}+ \beta_{2} X_{it}+ \boldsymbol{\beta}_{\boldsymbol{4}} \boldsymbol{Z}_{\boldsymbol{it}} + \theta_{t}+ {\alpha_{i}+ \varepsilon}_{it}$$

where the dependent variable $Y_{it}$ represents intrahousehold decision-making power, freedom of movement, or access to economic resources for three separate regression models of AGYW *i* at time *t* of married AGYW *i* at time *t (all AGYW were married at t = 1)*;

$X_{it}$ takes the value 1 if AGYW has ever experienced a live childbirth for an adolescent *i* at time *t*, and 0 otherwise;

$\boldsymbol{Z}_{\boldsymbol{it}}$ is a vector of time-varying controls that includes educational attainment, mass media exposure, wealth index, and household size;

$\theta_{t}$is a fixed effect for time (operationalized via a dummy variable for the second wave) and takes the value 1 if t = 2 and 0 otherwise;

$\alpha_{i}$ is a fixed subject-specific effect for adolescent *i;*

$\varepsilon_{it}$ is a random error term that follows a normal distribution with mean 0 and constant variance.

**Equation used to estimate association between the transition to becoming mother of son and changes in empowerment.**

We estimate the parameter of interest using an. For this, we use the following specification:

$$Y_{it}=\beta_{1}+\beta_{2} D_{it}+{\beta_{3} S}_{it} + \boldsymbol{\beta}_{\boldsymbol{4}}\boldsymbol{.}\boldsymbol{Z}_{\boldsymbol{it}} + \theta_{t}+ {\alpha_{i}+ \varepsilon}_{it}$$

where again our dependent variable $Y_{it}$ represents intrahousehold decision-making power, freedom of movement, or access to economic resources of adolescent *i* at time *t* for each specific regression model;

$D_{it}$ is an indicator variable that takes the value 1 if AGYW only had daughters, and 0 otherwise (at wave t=1 and if AGYW had no children or at least one son at wave t=2) for a given AGYW *i* at time *t*;

$S_{it}$ is an indicator variable that takes the value 1 if AGYW had at least one son, and 0 otherwise (at wave t=1 and if AGYW had no children or daughters only at wave t=2) for a given adolescent *i* at time *t;*

$\boldsymbol{Z}_{\boldsymbol{it}}$ is a vector of time-varying controls that includes educational attainment, mass media exposure, wealth index, and household size as discussed earlier;

$\theta_{t}$is a fixed effect for time (operationalized via a dummy variable for the second wave) and takes the value 1 if t = 2 and 0 otherwise;

$\alpha_{i}$ is a fixed subject-specific effect for AGYW *i;*

$\varepsilon_{it}$ is a random error term that follows a normal distribution with mean 0 and constant variance.

After estimating the model, we used the *lincom* command in Stata to estimate the additional effect of having had a son compared to having only daughters on empowerment of AGYW. The additional effect is given by the difference ($\hat{\beta})$ between the coefficients of $S_{it}$ and $D_{it}$ .
