# Supplementary material for: Role of marriage, motherhood, son preference on adolescent girls’ and young women’s empowerment: Evidence from a panel study in India
Source: PLoS One. 2023 Sep 28;18(9):e0292084. doi: 10.1371/journal.pone.0292084 (PMC10538655; doi:10.1371/journal.pone.0292084)
Supplement: Attachment — Submitted filename: PONE-D-23-10607_reviewer_REVIEWED.pdf [file pone.0292084.s004.pdf]

PLOS ONE

Role of marriage, motherhood, son preference on adolescent girls' empowerment: Evidence from a panel study in India

--Manuscript Draft--

**Manuscript Number:**	PONE-D-23-10607
**Article Type:**	Research Article
**Full Title:**	Role of marriage, motherhood, son preference on adolescent girls' empowerment: Evidence from a panel study in India
**Short Title:**	Role of marriage, motherhood and son preference on adolescent girls' empowerment in India
**Corresponding Author:**	Lakshmi Gopalakrishnan<br>University of California Berkeley School of Public Health<br>UNITED STATES
**Keywords:**	women's agency, women's empowerment, developing countries; South Asia
**Abstract:**	Background: Marriage is a key determinant of health and well-being of adolescent girls and young women (AGYW) in India. It is a key life event in which girls move to their marital households, often co-residing with their in-laws and begin childbearing. The change in the normative environment in conjunction with cultural norms surrounding son preference influences women's overall life course. However, there is scant research about the association between these life transitions and changes in empowerment among AGYW in India.<br>Methods: Using two waves of data from prospective cohort panel dataset that followed unmarried (6,065 observations in each wave) and married AGYW (3,941 observations from each wave) over a three-year period from Uttar Pradesh and Bihar, we examined how marriage, childbearing, and having a son is associated with changes in AGYW's empowerment, especially considering whether AGYW marry into patrilocal households (household with in-laws) as an effect modifier. Empowerment indicators included freedom of movement or mobility, decision-making power, access to economic using Kabeer's framework as our theoretical approach.<br>Results: Marriage was associated with lower freedom of movement with a pronounced effect on those who co-resided with their in-laws. Marriage was associated with greater decision-making power for AGYW who did not co-reside with the in-laws. Motherhood was positively correlated with greater freedom of movement, marginally higher intrahousehold decision-making power, and better access to economic resources. No statistically significant evidence that having at least one son compared to having daughters only (or no daughters) conferred additional changes in girls' freedom of movement, intrahousehold decision-making power, and access to economic resources.<br>Conclusion: Findings highlight the importance of understanding the vulnerabilities of being newly married in adolescence and emphasize the need for having interventions that target newly married AGYW along with mothers-in-law to empower them.
**Order of Authors:**	Lakshmi Gopalakrishnan
	Stefano Bertozzi
	Sophia Rabe-Hesketh
**Additional Information:**	
**Question**	**Response**
**Financial Disclosure**<br><br>Enter a financial disclosure statement that describes the sources of funding for the work included in this submission. Review the submission guidelines for detailed requirements. View published research	The authors received no specific funding for this work.

Powered by Editorial Manager® and ProduXion Manager® from Aries Systems Corporation

articles from *PLOS ONE* for specific examples.<br><br>This statement is required for submission and **will appear in the published article** if the submission is accepted. Please make sure it is accurate.<br><br>**Unfunded studies**<br>Enter: *The author(s) received no specific funding for this work.*<br><br>**Funded studies**<br>Enter a statement with the following details:<br>• Initials of the authors who received each award<br>• Grant numbers awarded to each author<br>• The full name of each funder<br>• URL of each funder website<br>• Did the sponsors or funders play any role in the study design, data collection and analysis, decision to publish, or preparation of the manuscript?<br>• **NO** - Include this sentence at the end of your statement: *The funders had no role in study design, data collection and analysis, decision to publish, or preparation of the manuscript.*<br>• **YES** - Specify the role(s) played.<br><br>* typeset	
**Competing Interests**<br><br>Use the instructions below to enter a competing interest statement for this submission. On behalf of all authors, disclose any competing interests that could be perceived to bias this work—acknowledging all financial support and any other relevant financial or non-financial competing interests.<br><br>This statement is required for submission and **will appear in the published article** if the submission is accepted. Please make sure it is accurate and that any funding sources listed in your Funding Information later in the submission form are also declared in your Financial Disclosure statement.	The authors have declared that no competing interests exist.

View published research articles from *PLOS ONE* for specific examples.<br><br>**NO authors have competing interests**<br><br>Enter: *The authors have declared that no competing interests exist.*<br><br>**Authors with competing interests**<br><br>Enter competing interest details beginning with this statement:<br><br>*I have read the journal's policy and the authors of this manuscript have the following competing interests: [insert competing interests here]*<br><br>* typeset	
**Ethics Statement**<br><br>Enter an ethics statement for this submission. This statement is required if the study involved:<br><br>• Human participants<br>• Human specimens or tissue<br>• Vertebrate animals or cephalopods<br>• Vertebrate embryos or tissues<br>• Field research<br><br>Write "N/A" if the submission does not require an ethics statement.<br><br>General guidance is provided below. Consult the submission guidelines for detailed instructions. **Make sure that all information entered here is included in the Methods section of the manuscript.**	N/A

Powered by Editorial Manager® and ProduXion Manager® from Aries Systems Corporation

**Format for specific study types**<br><br>**Human Subject Research (involving human participants and/or tissue)**<br>• Give the name of the institutional review board or ethics committee that approved the study<br>• Include the approval number and/or a statement indicating approval of this research<br>• Indicate the form of consent obtained (written/oral) or the reason that consent was not obtained (e.g. the data were analyzed anonymously)<br><br>**Animal Research (involving vertebrate animals, embryos or tissues)**<br>• Provide the name of the Institutional Animal Care and Use Committee (IACUC) or other relevant ethics board that reviewed the study protocol, and indicate whether they approved this research or granted a formal waiver of ethical approval<br>• Include an approval number if one was obtained<br>• If the study involved *non-human primates*, add *additional details* about animal welfare and steps taken to ameliorate suffering<br>• If anesthesia, euthanasia, or any kind of animal sacrifice is part of the study, include briefly which substances and/or methods were applied<br><br>**Field Research**<br><br>Include the following details if this study involves the collection of plant, animal, or other materials from a natural setting:<br>• Field permit number<br>• Name of the institution or relevant body that granted permission	
**Data Availability**<br><br>Authors are required to make all data underlying the findings described fully available, without restriction, and from the time of publication. PLOS allows rare exceptions to address legal and ethical concerns. See the PLOS Data Policy and FAQ for detailed information.	Yes - all data are fully available without restriction

A Data Availability Statement describing where the data can be found is required at submission. Your answers to this question constitute the Data Availability Statement and **will be published in the article**, if accepted.<br><br>**Important:** Stating 'data available on request from the author' is not sufficient. If your data are only available upon request, select 'No' for the first question and explain your exceptional situation in the text box.<br><br>Do the authors confirm that all data underlying the findings described in their manuscript are fully available without restriction?	
**Describe where the data may be found in full sentences. If you are copying our sample text, replace any instances of XXX with the appropriate details.**<br><br>• If the data are **held or will be held in a public repository**, include URLs, accession numbers or DOIs. If this information will only be available after acceptance, indicate this by ticking the box below. For example: *All XXX files are available from the XXX database (accession number(s) XXX, XXX.*).<br>• If the data are all contained **within the manuscript and/or Supporting Information files**, enter the following: *All relevant data are within the manuscript and its Supporting Information files.*<br>• If neither of these applies but you are able to provide **details of access elsewhere**, with or without limitations, please do so. For example:<br><br>*Data cannot be shared publicly because of [XXX]. Data are available from the XXX Institutional Data Access / Ethics Committee (contact via XXX) for researchers who meet the criteria for access to confidential data.*<br><br>*The data underlying the results presented in the study are available from (include the name of the third party*	The data that support the findings of this study are openly available in Harvard Dataverse at https://doi.org/10.7910/DVN/RRXQNT (Council, 2018)

and contact information or URL).<br>• This text is appropriate if the data are owned by a third party and authors do not have permission to share the data.<br><br>* typeset	
Additional data availability information:	

Powered by Editorial Manager® and ProduXion Manager® from Aries Systems Corporation

Manuscript Click here to access/download;Manuscript;Manuscript FULL.docx

Role of marriage, motherhood, son preference on adolescent girls' empowerment: Evidence from a panel study in India

Authors: Lakshmi Gopalakrishnan[1,2], Stefano Bertozzi[3], Sophia Rabe-Hesketh[4]

[1] Department of Health Policy and Management, 2121 Berkeley Way #5437, Berkeley, CA 94720, USA

[2] Present address: Institute for Global Health Sciences, University of California, San Francisco, 550 16th Street, 3rd Floor, San Francisco, CA 94158 lakshmi.gopalakrishnan2@ucsf.edu

[3] Department of Health Policy and Management, 2121 Berkeley Way #5437, Berkeley, CA 94720, USA sbertozzi@berkeley.edu

[4] School of Education, School of Education, UC Berkeley, 2121 Berkeley Way, Berkeley, CA 94720, USA sophiarh@berkeley.edu

Corresponding Author: Lakshmi Gopalakrishnan lakshmi.gopalakrishnan2@ucsf.edu
Institute for Global Health Sciences, University of California, San Francisco, 550 16th Street, 3rd Floor, San Francisco, CA 94158, Phone: +1-919-699-4346

Abstract

Background: Marriage is a key determinant of health and well-being of adolescent girls and young women (AGYW) in India. It is a key life event in which girls move to their marital households, often co-residing with their in-laws and begin childbearing. The change in the normative environment in conjunction with cultural norms surrounding son preference influences women's overall life course. However, there is scant research about the association between these life transitions and changes in empowerment among AGYW in India.

Methods: Using two waves of data from prospective cohort panel dataset that followed unmarried (6,065 observations in each wave) and married AGYW (3,941 observations from each wave) over a three-year period from Uttar Pradesh and Bihar, we examined how marriage, childbearing, and having a son is associated with changes in AGYW's empowerment, especially considering whether AGYW marry into patrilocal households (household with in-laws) as an effect modifier. Empowerment indicators included freedom of movement or mobility, decision-making power, access to economic using Kabeer's framework as our theoretical approach.

Results: Marriage was associated with lower freedom of movement with a pronounced effect on those who co-resided with their in-laws. Marriage was associated with greater decision-making power for AGYW who did not co-reside with the in-laws. Motherhood was positively correlated with greater freedom of movement, marginally higher intrahousehold decision-making power, and better access to economic resources. No statistically significant evidence that having at least one son compared to having daughters only (or no daughters) conferred additional changes in

girls' freedom of movement, intrahousehold decision-making power, and access to economic resources.

Conclusion: Findings highlight the importance of understanding the vulnerabilities of being newly married in adolescence and emphasize the need for having interventions that target newly married AGYW along with mothers-in-law to empower them.

Keywords:

women's agency, women's empowerment, developing countries; South Asia

Background

Marriage is a determinant of the health and well-being of adolescent girls and young women (AGYW) aged 15-24 years and their children in low-and-middle income countries (1,2). In patrilocal societies such as India, the cultural practice in which women move from a natal household to their marital household puts them in a new unfamiliar social and household environment (3). Women tend to lose ties with their natal kin and move to a household where they have subordinate status to both men and senior women of the household. Senior women, especially the mother-in-law, have control over household resources (4–6). Further, getting married in adolescence is associated with interruption of schooling, early childbearing, loss of peer networks, fewer economic opportunities, and poor health including increased risk of sexually transmitted diseases and high maternal mortality (7). The ability to pursue higher education, delay childbirth, or be employed depends on the wishes of husbands and in-laws, depriving newly married women of critical choices about their lives and well-being (8,9).

The next transition that affects young women is childbearing, which often comes immediately after marriage in South Asia, especially among lower socio-economic strata (10). About half of all pregnancies in India are unintended, with young women below 24 years having the highest rate of unintended pregnancies (10). Evidence from India demonstrates that young married women often do not have decision-making power to use contraception (11) and lack sexual agency (12). Early childbearing is detrimental to women and their children's nutritional status, healthcare utilization, and iron levels (13–15). Evidence also suggests that early marriage and pregnancy can adversely impact women's education and employment later in life (16).

Intertwined with giving birth in South Asia is gender bias at birth. Son preference is one example of deep-rooted culture-specific gender norms that result in health inequities for girls and women. Women are subjected to extreme social pressure to bear a son and undergo repeated pregnancies until a son is born (17–19). In patrilocal-patrilineal societies, sons are perceived to have greater economic, social, and religious utility compared to daughters because sons (or other male descendants) carry forward the family's name and legacy and inherit family assets, perform religious duties, and co-reside with their elderly parents caring for them during their old age (20). A prior study that leveraged several rounds of India’s Demographic Health Survey data suggested that mothers (15-49 years) who have a first-born daughter versus a son exhibit higher fertility, shorter birth spacing, and a greater risk of anemia with these outcomes worsening with successive daughters (female births) (21). Yet, another panel study that followed women aged 15-49 years over six years found no differential impact of having sons compared to daughters on mother’s status in the household measured using freedom of movement, household decision-

making, access to bank account, and ownership of assets (22). Overall, there is mixed empirical evidence on the differential impact of having sons on women's empowerment and status. Finally, no study to date from India has focused on studying son preference and empowerment measures among AGYW.

Life events such as marriage and childbirth may have an influence on empowerment as they shift household dynamics and change the life trajectory of women. However, prior empirical work examining the effects of marriage and childbirth on women's empowerment has been largely cross-sectional, with empowerment being assessed as a static measure. A limitation of this approach is that it does not acknowledge empowerment as a dynamic process in which prior and present life experiences may influence empowerment later. This study addressed these gaps by using a dataset that followed a panel of unmarried and married AGYW over a three-year period. We examined how marriage, childbearing, and specifically bearing a son affects changes in AGYW's empowerment in the early part of married life in India.

Women's empowerment

Women's empowerment is a multidimensional and complex construct. In this paper, we used Kabeer's definition of women's empowerment: "The expansion of people's ability to make strategic life choices in a context where this ability was previously denied to them" (23). This definition also fits with the broader concept of empowerment as defined in the World Bank's empowerment sourcebook: "the expansion of choice and action to shape one's life" (24). In both these definitions, the notion of empowerment stems from the state of disempowerment. Empowerment differs from related concepts of gender equality or women's autonomy through two distinct attributes. The first attribute is the explicit focus on the *processes of change* through

which women can achieve greater equality or greater choice. The second attribute is *agency* that puts women as central actors in the process of change (23,25). This ability to exercise choice further helps women make second-order choices that may be consequential to their quality of life, health, nutrition, and overall well-being.

Theoretical framework

We draw upon Kabeer's seminal framework for women's empowerment that underscores women's choice as essential to their power in the household and family (23).

In Kabeer's framework, three interrelated components of empowerment determine women's choices as outlined in *Figure 1*. The first component is resources that enable women to develop a capacity to articulate their preferences. These include human resources such as educational attainment, life skills, self-efficacy; economic resources such as paid work, earnings, bank accounts, property ownership; and social resources such as access to peer networks, role models, and connections that allow women to improve their situation beyond what is possible alone. The second component of the framework is agency defined as "the ability to define one's goals and act upon them" (23). Resources help women develop agency that comprises women's ability to make household decisions, freedom to move outside the house, reproductive and sexual decisions, bargaining power, and even cognitive processes such as reflection and analysis (23). Factors such as education, employment, marriage, and childbearing can enhance or deny women's agency (23). The third component is achievements that are outcomes achieved through increased access to enabling resources and enhanced agency. Given the interrelatedness of these three components, achievements of a particular moment can be transformed into enhanced resources and agency for a later time, improving women's capacity to make choices in the future (23). In summary, women's empowerment is a dynamic process, in which women acquire

human, social, and economic resources that enable women to gain agency to achieve their desired outcomes or aspirations.

Women's empowerment is not unitary, it has many broad dimensions in women's lives including economic, socio-cultural, political, legal, and psychological empowerment (25). Based on the measures available in our data, we used economic and socio-cultural empowerment measures in our study across two time points to examine empowerment as a dynamic process. We operationalized economic empowerment using indicators such as bank account ownership, having savings in the bank accounts, and ability to operate bank accounts. In our framework, economic empowerment indicators relate to enabling resources. For socio-cultural empowerment, we relied on two measures of women's empowerment that have been extensively used in the literature —women's participation in household decision-making and freedom of movement (26).

Extensive global evidence, including from South Asia, suggests that women's empowerment is associated with delayed initiation of childbearing, lower total fertility, greater birth spacing, higher contraceptive usage among women, and increased access to maternal health care (26–29), however, little is known about how marriage and fertility shape adolescent girls' and young women's (AGYW) empowerment. Examining how women's empowerment is linked with maternal and child health outcomes has been of interest to researchers trying to improve gender equality and women's health. We argue that studying women's empowerment should be an end goal because it impacts women's longer-term status and life opportunities.

Research on marriage and fertility in the South Asian context has been somewhat narrow in scope due to the paucity of panel data that longitudinally follow AGYW over time to examine the transitions in empowerment associated with life events. In a setting like India, the median age

at first marriage is 19 years among women aged 20-49 years. 27% of women aged 20-24 years had a "child marriage," i.e., married before they were 18 years old (10). Childbearing often begins soon after marriage—about 60% of women under 25 were pregnant within the first year of marriage. Women in India spend most of their lifetime in the institution of marriage (10). It is essential to study the trajectory of AGYW empowerment through their life course especially as they transition into marriage and start their reproductive journey. Given that childbearing occurs mostly early in marriage, its impact on women's empowerment may set the stage for the rest of their life in their marital household.

We used a panel dataset that followed unmarried and married AGYW, allowing us to examine how marriage and childbearing shape AGYW's empowerment in the early part of their married life. Using Kabeer's framework, empowerment was examined as a dynamic process over AGYW's life course by studying the immediate changes to empowerment resulting from marriage and attaining motherhood status. Further, given the prevalence of son preference in the Indian context, we also studied whether having sons conferred additional advantages to AGYW's (i.e., mother's) empowerment compared to having only daughters.

Research Aims

The focus of this paper is on the following research questions and corresponding hypotheses:

(1) Is marriage associated with a change in empowerment of AGYW? Further, does the relationship between marriage and empowerment depend on whether the household arrangement is patrilocal (newly married women co-residing with in-laws)? Is marriage into a patrilocal household (co-residence with in-laws) associated with a greater change in empowerment for AGYW compared to marriage into a household without in-laws?

Hypothesis 1A: Getting married will be associated with a decrease in empowerment (freedom of movement, decision-making power, and access to economic resources) of AGYW

Hypothesis 1B: Given the prior evidence on patrilocal marriages (co-residence with in-laws), AGYW married into a living arrangement of co-residence in-laws will have a greater decrease in empowerment compared to AGYW married into a living arrangement without in-laws.

(2) Is motherhood associated with a change in empowerment of married AGYW?

Hypothesis 2: Considering the importance of women's fertility in the Indian context, we hypothesized that transitioning to motherhood will lead to a greater increase in empowerment across all dimensions.

(3) Is having a son associated with a greater change in empowerment among married AGYW compared to having daughters only?

Hypothesis 3: Given the long-standing social norms around son preference in the Indian context, we hypothesized that AGYW who have had at least one son will have a greater increase in empowerment compared to those AGYW who only have daughters.

Methods

Data

We used a prospective longitudinal cohort dataset of adolescents from Population Council's UDAYA (Understanding Lives of Adolescents and Young Adults) study, which was conducted in two large northern states—Bihar and Uttar Pradesh (UP)—in India (30). Two waves of panel data were collected: Wave 1 in 2015-16 and Wave 2 in 2018-19. Wave 1 surveyed 20,594 unmarried boys and girls aged 10-19 and married girls aged 15-19 from a state-representative

random sample of 300 urban and rural primary sampling units (PSUs)—villages in rural areas and census wards in urban areas. To answer our study questions, we used a sample of unmarried and married AGYW (15-19 years) at Wave 1 and those followed up at Wave 2 when they turned 18-22 years. Of the 7,776 unmarried AGYW (15-19 years) interviewed in Wave 1, the team re-interviewed 6,168 AGYW (18-22 years) in Wave 2 with a re-contact rate of 79%. Of the 5,206 married AGYW (15-19 years) interviewed in Wave 1, the team re-interviewed 4,257 AGYW (18-22 years) in Wave 2 with a re-contact rate of 82%. The re-contact rates of 79% and 82% in both these samples meet the criteria of minimum acceptable re-contact in longitudinal studies (31).

The UDAYA study was a multi-topic survey that covered different modules related to demographics, economic activity, household work and migration, mass media and social media exposure, social and peer environment, aspirations, gender role attitudes, awareness of sexual and reproductive health matters, empowerment measures, transitions to marriage and parenthood. The study received ethical approval from the Institutional Review Boards of Population Council USA and Centre for Media Studies, India. Since we used de-identified data publicly available from the Harvard Dataverse website, we were exempt from seeking ethical approvals (32). We did not have access to identifying individual participant data.

Our analytical sample was restricted to the panel of AGYW who were unmarried at Wave 1 and followed up at Wave 2 to answer the first research question. Given that we are interested in understanding the relationship between transition to marriage and empowerment, we dropped adolescent girls who were married but had separated or divorced between Wave 1 and Wave 2 (n=27). We recoded the adolescent girls who were married as unmarried if they were married but

had not had *gauna*[1] (effectively had not moved to their husbands' home to start a marital life) (n=26) and dropped observations where the household details were not collected at the second wave (n=24). Our final analytical sample for this question used a balanced panel of 12,130 observations (6,065 observations in each wave).

We used the sample of married adolescents who were re-contacted again to answer the second and third research questions. Of the 4,257 girls surveyed at Wave 1, 257 adolescent girls had still not performed *gauna*. Of the 4,000 adolescent girls who were married and cohabiting with their husbands at Wave 1, 35 were divorced or separated by Wave 2 and dropped observations where the household details were not collected at the second wave (n=24). Hence, we have a sample of 3,941 adolescent girls who were already married in 2015-16, still married in 2018-19, and cohabiting with their husbands at both waves. Our paper uses a balanced panel of 7,882 observations (3,941 observations from each wave). We had comparable measures for AGYW across the two waves, including repeated empowerment measures: decision-making, freedom of movement, and access to economic resources.

Variable definitions and operationalization

Dependent variables:

We used three measures to capture different dimensions of AGYW's empowerment: (i) freedom of movement (or mobility), (ii) decision-making power, both of which reflect AGYW's agency,

---

[1] Gauna is a custom in northern India and the ceremony associated with the consummation of marriage often associated with early marriage of girls. Marriage is considered as a ritual union with conjugal life beginning after gauna. Until gauna is performed, married girls stay in their natal home.

and (iii) access to economic resources, which captures AGYW's access to enabling resources (Figure 1). The three main dependent variables of empowerment are further detailed below:

(1) Freedom of movement or mobility: The survey collected information on whether and how the AGYW travelled to three types of places: (i) a shop or market or a friend/relative's place *inside* their village/ward, (ii) a shop or market or a friend/relative's place *outside* their village/ward, and (iii) to attend any program (health event, girls meeting, sports event) in their village/ward. For each of these questions, we assigned a score of 2 if girls were allowed to go alone, 1 if they were allowed to go but only with a companion, and 0 if they were not allowed to go out at all. Responses were summed to create a scale (0 to 6) with higher responses indicating a higher amount of freedom in mobility.

(2) Decision-making power: AGYW were asked who mainly decides about: (i) how much education the respondent should have, (ii) making major household purchases, and (iii) whether the respondent should work or stay at home. For each of these variables, we assigned a score of 2 if the respondent reported making decisions solely, 1 if the respondent takes decisions jointly with others and 0 if the respondent reported having no say in making decisions. Items were summed to form a scale (0 to 6) with higher scores indicating greater decision-making ability of the respondent.

(3) Access to economic resources: Three survey questions were included on whether girls had their own bank account (*yes=1, no=0*), whether they operated their own account (*yes=1, no=0)*, and whether they had savings in their bank accounts (*yes=1, no=0)*.

Responses were summed to create a scale (0 to 3) with higher responses indicating a greater access to economic resources.

Independent variables:

(1) Marriage: An indicator variable for whether the adolescent girl is married or unmarried as captured by the question to ascertain the adolescent's marital status (AGYW who were unmarried at wave 1).

(2) Motherhood: An indicator variable for whether the adolescent has experienced a live childbirth (AGYW who were married at wave 1).

(3) Having a son: A categorical variable to indicate whether the AGYW has had daughters only, at least one son, or no children (reference category). This variable was used for the AGYW who were married at wave 1.

Covariates

Sociodemographic covariates that may be related to marriage, fertility, and empowerment were selected *a priori* based on prior literature to ensure that sociodemographic variables are not confounding the explanations for primary hypotheses. Past literature suggests that girls' education is associated with an increase in their empowerment, status, and intra-household bargaining power (22,33). Following Jensen's panel study which found that the introduction of satellite television was significantly associated with an increase in women's household decision-making and a decrease in her son preference, we included exposure to mass media as one of the controls (34). The role of household-level factors such as a household wealth index, the presence of in-laws in the marital household, and family type (nuclear versus joint household) have also been associated with women's empowerment (35–38). Household wealth index was computed using principal components analysis with a standard set of questions on a household's ownership

of consumer items such as a television, scooter and other assets, dwelling characteristics such as flooring material, type of drinking water source, and toilet facilities.

To understand the association between the transition to marriage and changes in empowerment, we controlled for time-varying covariates such as girls' education (as a continuous score) and exposure to mass media (television, radio, and newspaper). We also controlled for whether those who were married by the second wave had already *borne a child* to disentangle the independent effects of being married on changes in empowerment. To study the heterogeneous effects of the association between marriage and changes in empowerment, we included co-residence with in-laws as an effect modifier. The effect modifier, *co-residence with in-laws,* was only asked in the second wave of the UDAYA data. AGYW who were married into a household with in-laws were coded as 1, and 0 otherwise. Co-residence variable was coded as 0 in wave 1 for all AGYW and as 0 in wave 2 for AGYW who remained unmarried, so the coefficients can be interpreted as interaction with marital status. Finally, we also controlled for survey wave (Wave 2) because of the possibility of secular trends in empowerment over time.

Analysis

We used fixed effects modeling, specifically modeling the within-individual variation across the two survey waves, to examine how changes in an AGYW's marital status and motherhood status are associated with changes in their empowerment. A key strength of this approach is the ability to control for unobserved confounders at the individual level that do not change over time, removing much of the omitted variable bias. In our analyses, the "marriage effect" can be interpreted as the effect of marriage on changes in AGYW empowerment over time. Similarly, the "motherhood effect" can be interpreted as the effect of becoming a mother on changes in AGYW empowerment over time and the "mother of son effect" can be interpreted as the effect

of bearing a son compared to having daughters only or no children on changes in AGYW empowerment over time.

As a first step in our analyses, we generated descriptive statistics for all variables of interest, by wave of data collection, that allowed us to explore the characteristics of 15-19 year-old unmarried AGYW and married AGYW at both waves.

For the first question, we used individual fixed-effects regressions to capture how a change in marital status from being unmarried to married is associated with changes in the empowerment domains mentioned previously. Since all the dependent variables are continuous, we use a linear fixed-effects model on a balanced panel to analyze the association between marital status and empowerment over time using the specification below:

$$Y_{it} = \beta_1 + \beta_2 X_{it} + \beta_3 X_{it} \times I_{it} + \boldsymbol{\beta_4} \boldsymbol{Z_{it}} + \theta_t + \alpha_i + \varepsilon_{it}$$

Here Y_{it} represents one of the three dependent variables including intrahousehold decision-making power, freedom of movement, and access to economic resources of adolescent *i* at time *t* *(all AGYW were unmarried at t = 1)* for three separate regression models;

X_{it} represents the marital status of AGYW *i* at time *t (all AGYW were unmarried at t = 1)* for those married into a household without in-laws*;*

$X_{it} \times I_{it}$ takes the value 1 if AGYW gets married into a household with in-laws at the second wave *(t = 2)* and 0 otherwise (i.e., wave t=1, unmarried at wave t=2, or married into less wealthy household at wave t=2) for an AGYW *i* and represents the interaction term between marriage and co-residence with in-laws*;*

$\boldsymbol{Z_{it}}$ is a vector of time-varying AGYW-level characteristics such as educational attainment and mass-media exposure;

θ_t is a fixed effect for time (operationalized via a dummy variable for the second wave);

α_i is a fixed subject-specific effect for AGYW *i;*

ε_{it} is a random error term that follows a normal distribution with mean 0 and constant variance.

We also used the *lincom* command to calculate the effect of marriage on empowerment domains for the interaction term on those co-residing with in-laws compared to not co-residing with their in-laws.

To estimate the association between the transition to motherhood and changes in empowerment, we ran similar individual linear fixed-effects regressions on the sample of married AGYW for each of the domains of empowerment over time listed previously using the specification below:

$$Y_{it} = \beta_1 + \beta_2 X_{it} + \boldsymbol{\beta_4} \boldsymbol{Z_{it}} + \theta_t + \alpha_i + \varepsilon_{it}$$

where the dependent variable Y_{it} represents intrahousehold decision-making power, freedom of movement, or access to economic resources for three separate regression models of AGYW *i* at time *t* of married AGYW *i* at time *t (all AGYW were married at t = 1)*;

X_{it} takes the value 1 if AGYW has ever experienced a live childbirth for an adolescent *i* at time *t*, and 0 otherwise;

$\boldsymbol{Z_{it}}$ is a vector of time-varying controls that includes educational attainment, mass media exposure, wealth index, and household size;

θ_t is a fixed effect for time (operationalized via a dummy variable for the second wave) and takes the value 1 if t = 2 and 0 otherwise;

α_i is a fixed subject-specific effect for adolescent *i;*

ε_{it} is a random error term that follows a normal distribution with mean 0 and constant variance.

Finally, we examined the association between having at least one son and change in empowerment by comparing married AGYW who had a son (with or without daughters) with those who had daughters only or no children. We estimate the parameter of interest using an individual panel fixed-effect regression. For this, we use the following specification:

$$Y_{it} = \beta_1 + \beta_2 D_{it} + \beta_3 S_{it} + \boldsymbol{\beta_4} . \boldsymbol{Z_{it}} + \theta_t + \alpha_i + \varepsilon_{it}$$

where again our dependent variable Y_{it} represents intrahousehold decision-making power, freedom of movement, or access to economic resources of adolescent *i* at time *t* for each specific regression model;

D_{it} is an indicator variable that takes the value 1 if AGYW only had daughters, and 0 otherwise (at wave t=1 and if AGYW had no children or at least one son at wave t=2) for a given AGYW *i* at time *t*;

S_{it} is an indicator variable that takes the value 1 if AGYW had at least one son, and 0 otherwise (at wave t=1 and if AGYW had no children or daughters only at wave t=2) for a given adolescent *i* at time *t;*

$\boldsymbol{Z_{it}}$ is a vector of time-varying controls that includes educational attainment, mass media exposure, wealth index, and household size as discussed earlier;

θ_t is a fixed effect for time (operationalized via a dummy variable for the second wave) and takes the value 1 if t = 2 and 0 otherwise;

α_i is a fixed subject-specific effect for AGYW *i;*

ε_{it} is a random error term that follows a normal distribution with mean 0 and constant variance.

After estimating the model, we used the *lincom* command in Stata to estimate the additional effect of having had a son compared to having only daughters on empowerment of

AGYW. The additional effect is given by the difference $(\hat{\beta})$ between the coefficients of S_{it} and D_{it} .

Across all the models, we set the level of statistical significance (Wald p-value) at 0.05. Robust standard errors were used to relax assumptions of constant variance and constant within-subject residual correlations. All data were analyzed using Stata Version 15.1 (39).

Results

Characteristics of the sample

Table 1 (the first column) shows the demographic characteristics of unmarried AGYW (n=6,065) at Wave 1 across both states. At Wave 1, on average, unmarried AGYW were 16 years old. Approximately 6% of AGYW never received formal education, a little over half (55.9%) of AGYW completed some level of secondary education, and 32% of the AGYW completed some primary education. Less than one in five adolescent girls (18%) reported having undertaken paid work in the 12 months preceding the survey. Over half (55%) the girls lived in rural settings. A majority (72.9%) of adolescent girls reported being exposed to mass media, including radio, TV, and newspapers, at least once a month. One in five AGYW (21%) reported ever having received sexual and reproductive health information. Of the 6,065 unmarried girls, 24.4% (n=1482) got married in the interval between Wave 1 and Wave 2.

The second column of *Table 1* describes the demographics of married AGYW at Wave 1 (n=3941). At Wave 1, the average age of married AGYW is 18 years. Unlike the cohort of unmarried AGYW, a little over a quarter (27%) of the married AGYW did not receive any formal education, 37.6% completed some primary education and about a third (32.1%) completed some secondary education. Only one in ten AGYW (10.7%) reported doing paid work recently in the 12 months preceding the survey. Sixty-three percent of married AGYW lived in

rural settings. Less than half (48.8%) of married AGYW girls reported being exposed to mass media, including radio, TV, and newspapers, at least once a month. One in ten AGYW (10.9%) reported ever having received sexual and reproductive health information. Of the 3,941 married AGYW, 46% had begun childbearing by the first Wave of the survey.

Below we summarize the regression results.

Transition to marriage and empowerment domains

Table 2 presents the coefficients from the individual panel linear fixed effects regression. Controlling for having a childbirth by wave 2, girls' education, exposure to mass media, and time, we found that being married is associated with a reduction in mobility of AGYW ($\hat{\beta}$ = -0.45, 95%CI= -0.63, -0.27, p<0.001) for AGYW not marrying into household where they co-reside with in-laws. Co-residence with in-laws was a significant effect modifier of the relationship between marriage and freedom of movement ($\hat{\beta}$ = -0.87, 95%CI= -1.06, -0.69, p<0.001) holding other covariates constant. Specifically, AGYW who marry into a household with in-laws had a more pronounced decrease in freedom of movement compared to AGYW who married and did not co-reside with their in-laws ($\hat{\beta}$ = -1.13, 95%CI= -1.47, -1.20, p<0.001).

On average being married was associated with greater decision-making power ($\hat{\beta}$ = 0.29, 95%CI: 0.10, 0.47, p=0.002) for AGYW who did not marry into a household where they co-reside with their in-laws, holding all else constant. Marrying into a household with in-laws is an effect modifier in the relationship between marriage and decision-making power ($\hat{\beta}$ = -0.39, 95%CI: -0.57, -0.21, p=0.002). Specifically, we did not observe a statistically significant association between marriage and decision-making power among AGYW who marry into a household with in-laws compared to AGYW who were married without co-residence with in-laws ($\hat{\beta}$ = -0.10, 95%CI: -0.25, 0.04, p=0.174).

We also found that on average, there was a positive association between marriage and increased access to economic resources ($\hat{\beta}$ = 0.37, 95%CI= 0.23, 0.51, p<0.000) for AGYW not marrying into household where they co-reside with in-laws. We did not find that the association between marriage and access to economic resources varied by living arrangement (co-residence with in-laws).

Association between motherhood status and empowerment domains

Table 3 depicts the results from individual fixed effects regressions examining the association of motherhood status and empowerment domains among the panel of married AGYW. Becoming a mother is associated with an increase in mobility of AGYW ($\hat{\beta}$ = 0.12, 95%CI= 0.02, 0.23, p = 0.023). We did not find a positive association between motherhood and intrahousehold decision-making power of AGYW ($\hat{\beta}$ = 0.09, 95%CI= -0.00, 0.19, p=0.058) controlling for other variables and time (wave) in the model. Further, becoming a mother was also positively correlated with higher access to economic resources ($\hat{\beta}$ = 0.24, 95%CI = 0.16, 0.32, p < 0.001) holding other variables constant.

Estimating the role of having sons on AGYW empowerment

Based on the models below in *Table 4*, holding all controls and time constant, we find evidence that having daughters only (compared with no children) is positively associated with access to economic resources. Similarly, having at least one son (compared with no children) is associated with greater freedom of movement and greater access to economic resources of AGYW. However, there is no evidence of an additional effect of having a son over and above the effect of having daughters. In other words, there is no differential change in empowerment between AGYW who have borne at least one son compared to having only daughters.

Discussion

To our knowledge, this is the first panel study of adolescents and young women (AGYW) from India to explore the changes in their social and economic empowerment associated with critical life events such as marriage, motherhood, and son preference. By leveraging data from unmarried and married AGYW across two time points, we examined empowerment as a dynamic process, overcoming a limitation of prior cross-sectional studies that examine empowerment as a static measure. Drawing on Kabeer's seminal framework, we examined how marriage and motherhood are associated with measures of AGYW's agency and enabling resources. Specifically, we tested whether marrying into a patrilocal household (i.e., co-residence with in-laws) moderates the association between marriage and empowerment. Finally, we examined the effects of motherhood on empowerment and studied the effects of becoming a mother of a son over having only daughters (or no daughters) using the married AGYW dataset.

As hypothesized, our results showed that on average, marriage was associated with lower freedom of movement among AGYW controlling for covariates and time. We also found that married AGYW co-residing with their in-laws had a much lower freedom of movement compared to AGYW who do not co-reside with their in-laws. These results are consistent with prior evidence from India suggesting that co-residence with the mother-in-law was negatively associated with the daughter-in-law's mobility (40,41) and ability to form social connections (37). Living with in-laws means more hierarchy within the household that tends to decrease the empowerment of the youngest daughters-in-law (8,37,42). Newly married women (daughters-in-law) often have the lowest status in the household compared to men and other senior women in the household especially during the early period of their marriage (4,5). For instance, daughters-

in-law are often expected to follow gendered norms of eating last in the household and are socially isolated from their peer networks (7,37,43). Apprehensions about the use of contraception, influence of outside peers on daughters-in-law, and fear of losing authority can potentially explain the negative effects on mobility of daughters-in-law (37).

We found that marriage was associated with greater decision-making power for AGYW who did not co-reside with the in-laws. However, we did not observe a statistically significant association between marriage and decision-making power among those who co-resided with their in-laws compared to those who did not co-reside with their in-laws. This finding contradicts results from an Indian cross-sectional study that noted that compared to women married into nuclear families, women ages 15-49 years married in extended family households living with in-laws had lower decision-making power (8,40,41).

As hypothesized, we found that motherhood was positively correlated with greater freedom of movement, marginally higher intrahousehold decision-making power, and better access to economic resources. Our results are consistent with prior evidence from another panel study from India that found that motherhood was associated with increased freedom of movement and gaining access to a bank account (22). Another study using retrospective data from a northern state in India also demonstrated that women in childbearing stages had higher agency and less fear of spousal violence than those who had not yet started childbearing (44).

Our results of increased empowerment among young mothers are not entirely surprising because women's status is inherently tied to their fertility. However, this increase in women's empowerment may come at the expense of in-laws' pressures to bear children immediately after marriage, potentially reflecting the limited ability of women to control their fertility (45). In an ethnographic study of young Nepali women, some young women reported feeling uncomfortable

asserting their agency out of a desire to conform to being a "good woman and daughter-in-law" (46). Such forms of "strategizing" by women in the 'classic patriarchal belt' (from North Africa and Middle East to South Asia) has been given the term "patriarchal bargain", in which young brides accept "hardship" as a tradeoff to gain economic security through their spouse (6). Our results also complement the research carried out in other contexts where women's value is linked to their fertility such as the Middle East (47). In addition to the norms surrounding fertility, childbearing brings with it practices surrounding childcare that might offer the women opportunities to step outside their homes, seek healthcare for their children, and make decisions within the household. One interpretation for increased access to economic resources could be due to conditional cash transfer programs being targeted at pregnant women to increase institutional births, expanding women's access to bank accounts and savings (48).

Contrary to our hypothesis, we did not find statistically significant evidence that having at least one son compared to having daughters only (or no daughters) conferred additional changes in girls' freedom of movement, intrahousehold decision-making power, and access to economic resources. Overall, these findings are consistent with another study that examined motherhood effects on a panel of Indian women (15-49 years) that did not find that motherhood effects on empowerment measures were different by sex of child (22). However, our results deviated from two cross-sectional studies in which authors found that having more sons was associated with greater autonomy among women in Uttar Pradesh, India (35) and having a male first birth was associated with clean cooking fuels that authors assert could be due to greater intrahousehold bargaining power of women or elevated social status for women in urban India (49). One plausible explanation for non-differential changes in AGYW's empowerment by sex composition could be progressive gender policies and national girl child focused programs such

as *Beti Bachao Beti Padhao (*Save and Educate the Girl Child) that have been found to improve sex ratio at birth in one of the Indian states with the worst sex ratio at birth (50,51). Further, there has been an improvement in sex ratio at birth in both the study states (Bihar and UP), potentially reflecting a broader societal change in attitudes towards girl children (10). Finally, since the participants in our study are young and may continue childbearing, more rounds of data collection in the future may allow us to continue examining differential effects on the trajectory of empowerment by sex composition of children.

Despite making some significant contributions to the literature, we acknowledge that our study has some limitations. First, we studied only two northern states in India which are similar in social and cultural norms surrounding women's status and role in the society and thus our results are not generalizable to other regions of India. Second, the data were collected in two waves in 2015-16 and 2018-19. This three-year duration between the two waves could possibly be insufficient to capture the longer-term effects of marriage and childbearing on domains of empowerment including mobility, intrahousehold decision-making, and access to economic resources from a lifecourse perspective. While we were able to study immediate changes in empowerment following marriage and childbirth, we were not able to study lifetime influences on the process of empowerment. Further, the empowerment measures we used capture only the economic and socio-cultural dimensions of empowerment and do not capture other dimensions of empowerment such as political, legal, and psychological empowerment. Given the multidimensional nature of empowerment, empowerment in one domain may not necessarily translate into empowerment in other domains (25,52,53). The analysis was also limited in its ability to unpack the mechanisms through which marriage and childbirth are associated with changes in empowerment over time.

Our study has many strengths worth noting. To our knowledge, this is one of the two studies in the Indian context that has examined the relationship between childbirth and empowerment and the first study that has longitudinally analyzed how the transition from being unmarried to married impacts AGYW's empowerment over time. Second, we were able to utilize this panel dataset that followed state-representative samples of unmarried and married AGYW at two time points to examine the process of women's empowerment immediately after their marriage and childbirth. In India, childbearing follows soon after marriage, hence by following recently married young women, we were able to study short-term changes in empowerment over time. We also were able to examine empowerment as a process over time by using panel fixed effects that remove the omitted variable bias arising from unobserved time-invariant variables. We also grounded our analysis in one of the seminal frameworks in the field of empowerment, whose underuse has been one of the drawbacks of empirical studies in the field of women's empowerment literature (53). By using within-person fixed effects analysis, any measurement error related to different cognitive understanding or semantic meaning of empowerment questions across individuals have been minimized (54). Third, our measures of empowerment are relevant to the context of North Indian settings and are also like the indicators measured in Demographic Health Surveys rendering it comparable. Finally, by controlling for wave (or time), we were able to control for secular trends and maturity the adolescents may acquire over age and time in their marriage.

Policy implications

Achieving gender equality and empowering all women and girls is the fifth goal in the 2030 agenda for the Sustainable Development Goals (SDGs)(55). Our findings on marriage and empowerment highlight the importance of understanding vulnerabilities associated with being

newly married during adolescence, particularly how limited freedom of movement and lower decision-making power could impede newly married adolescents' access to essential services, including family planning and other health-seeking behaviors. Our findings also highlight the need to have health interventions that target both daughters-in-law and mothers-in-law together. This is particularly important because in the Indian context childbearing follows immediately after marriage. Our findings also place importance on changing restrictive gendered cultural norms that put newly married women at the lowest status in the marital household. Empowering women has benefits not only for themselves, but also for their children as women's empowerment has been associated with many maternal and child health outcomes (29).

Childbearing was associated with greater empowerment across the dimensions chosen for the study. Findings underscore the high value placed on childbearing in this setting and re-emphasize the need to address harmful social and gender norms that define women's status within the confines of their reproductive potential. Helping shift restrictive gender norms so that the multitude of other contributions (female labor force participation, unpaid caregiving) that women make are more highly valued is essential.

Conclusion

In this study, we examined the association between transition to marriage and childbirth on social and economic dimensions of empowerment using a panel data from two large states of India. We extended the existing literature by investigating women's empowerment over time and whether marriage, motherhood, and bearing at least one son are associated with multiple types of AGYW empowerment in India. Findings emphasize the need for interventions that target newly married young women along with key household members such as mothers-in-law to empower

them. Taken together, our findings reiterate the notion that empowerment is a dynamic process impacted by life events such as marriage and childbirth.

Table 1: Characteristics of unmarried and married adolescent girls interviewed at Wave 1 (2015-16), UDAYA longitudinal survey

	**n (%) of AGYW-unmarried (n= 6,065) at Wave 1, 2015-16**	**n (%) of AGYW-married (n=3,941) at Wave 1, 2015-16**
**Age (mean ± SD)**	16.6 ± 1.3	18.0 ± 1.0
**Education**		
No formal education	384 (6.3%)	1,079 (27.3%)
Completed some primary education (1-8 Grade)	1,945 (32.0%)	1,480 (37.6%)
Completed some secondary education (9-12 Grade)	3,387 (55.9%)	1,263 (32.1%)
Completed some college education	349 (5.8%)	119 (3.0%)
**Household wealth index**		
Poorest	1,525 (25.1%)	991 (25.1%)
Poor	1,524 (25.1%)	981 (24.9%)
Rich	1,525 (25.1%)	993 (25.2%)
Richest	1,491 (24.7%)	976 (24.7%)
**Religion**		
Hindu	4,479 (73.9%)	3,336 (84.6%)
Muslim	1,562 (25.7%)	594 (15.0%)
Other	24 (0.4%)	11 (0.2%)
**Caste**		
Scheduled Caste / Scheduled Tribe	1,262 (20.8%)	1,181 (29.9%)
Other Backward Caste	3,444 (56.8%)	2,375 (60.2%)
General Caste	1,359(22.4%)	385 (9.7%)
**Recent paid work**		
No	4,975 (82.1%)	3,516 (89.3%)
Yes	1,090 (17.9%)	425 (10.7%)
**Household size (mean ± SD)**	6.5 ± 2.7	5.8 ± 3.0
**Exposure to mass media (TV, radio, newspaper) at least once a month**		
No	1,652 (27.2%)	2,018 (51.2%)
Yes	4,413 (72.7%)	1,923 (48.8%)
**Received sexual reproductive health education**		
No	4,741 (78.1%)	3,512 (89.1%)
Yes	1,324 (21.9%)	429 (10.9%)
**Residence**		
Urban	2,729 (45.0%)	1,428 (36.2%)
Rural	3,336 (55.0%)	2,513 (63.7%)
**State**		
Uttar Pradesh (UP)	3,315 (54.6%)	1,299 (32.9%)
Bihar	2,750 (45.4%)	2,642 (67.1%)
**Marital Status at Wave 1**		
Unmarried	6,065	-
Married	-	3,941
**Had a childbirth at Wave 1**		
No	6,065	2,128 (54.0%)
Yes	-	1,813 (46.0%)

Table 2: Regression coefficients from individual fixed effects regressions for the association between marriage and freedom of movement, decision-making power, access to economic resources among adolescent girls and young women (AGYW) in the two study states (n=12130)

**Marriage and empowerment**									
	Freedom of movement			Decision-making power			Access to economic resources		
	Coeff.	95%CI	p-value	Coeff.	95%CI	p-value	Coeff.	95%CI	p-value
**Being married**	-0.45	-0.63, -0.27	<0.000	0.29	0.10, 0.47	0.002	0.37	0.23, 0.51	<0.000
**Marriage X co-residence with in-laws (Ref: Co-residence with husband/other adults)**									
Being married and co-residing with in-laws	-0.87	-1.06, -0.69	<0.000	-0.39	-0.57, -0.21	<0.001	-0.08	-0.22, 0.06	0.256
**Controls**									
Having had a childbirth	0.15	-0.02, 0.31	0.086	-0.02	-0.18, 0.14	0.805	0.28	0.16, 0.41	<0.001
Educational attainment of AGYW	0.02	-0.01, 0.05	0.133	-0.06	-0.09, -0.02	<0.001	0.10	0.07, 0.12	<0.001
Exposure to mass media	0.10	0.02, 0.18	0.015	0.01	-0.07, 0.09	0.874	0.06	0.00, 0.13	0.037
Wave (Ref: Wave 1)									
Wave 2	0.32	0.26, 0.39	<0.000	0.08	0.01, 0.15	0.026	0.21	0.16, 0.26	<0.001
Mean (SD) for unmarried AGYW	4.6 (1.2)			1.9 (1.5)			1.7 (1.1)		
**Number of Observations**	12130			12130			12130		

Notes: [1] Husband/wife alone or with wife' parents or other relatives; Robust 95 per cent confidence intervals

Table 3: Regression coefficients from three individual fixed effects regressions for the association between motherhood and freedom of movement, decision-making power, and access to economic resources among married AGYW in India (n=7882)

**Motherhood and empowerment**									
	Freedom of movement			Decision-making power			Access to economic resources		
	Coeff.	95%CI	p	Coeff.	95%CI	p	Coeff.	95%CI	p
**Motherhood**	0.12	0.02, 0.23	0.023	0.09	-0.00, 0.19	0.058	0.24	0.16, 0.32	<0.001
**Controls**									
**Girls' education**	-0.05	-0.14, 0.04	0.271	-0.04	-0.13, 0.05	0.361	-0.19	-0.26, -0.12	<0.001
**Wealth index scores**	-0.03	-0.06, 0.00	0.076	-0.04	-0.07, -0.01	0.004	0.03	0.00, 0.05	0.027
**Household size**	-0.03	-0.04, 0.01	0.002	-0.04	-0.05, -0.02	<0.001	0.00	-0.02, 0.01	0.599
**Mass media exposure**	0.09	0.00, 0.19	0.048	0.09	0.00, 0.17	0.048	0.17	0.10, 0.24	<0.001
**Wave 2 (Ref: Wave 1)**	0.80	0.73, 0.87	<0.001	0.11	0.04, 0.17	0.001	0.55	0.49, 0.61	<0.001
**Average for girls without children**	3.2 (1.3)			1.6 (1.4)			1.2 (0.9)		
**Number of Observations**	7882			7882			7882		

Note: Robust 95 per cent confidence intervals.

Table 4: Regression coefficients from three separate individual fixed effects regressions for the association between sex composition of children and freedom of movement, decision-making power, and access to economic resources among adolescent girls and young women

**Mother of sons and empowerment**									
	Freedom of movement			Decision-making power			Access to economic resources		
	Coeff.	95%CI	p-value	Coeff.	95%CI	p-value	Coeff.	95%CI	p-value
**Having daughters** (Ref: no children)	0.10	-0.02, 0.22	0.094	0.10	-0.01, 0.20	0.075	0.23	0.14, 0.31	<0.001
**Having at least one son** (Ref: no children)	0.15	0.02, 0.27	0.020	0.09	-0.03, 0.20	0.134	0.25	0.16, 0.34	<0.001
**Additional effect of having a son compared to daughters only**	0.05	-0.07, 0.17	0.432	-.010	-0.11, 0.09	0.848	0.024	-0.07, 0.11	0.588
**Controls**									
**Girls' education**	-0.05	-0.14, 0.04	0.287	-0.04	-0.13, 0.05	0.357	-0.19	-0.25, -0.12	<0.001
**Wealth index**	-0.03	-0.06, 0.00	0.078	-0.04	-0.07, -0.01	0.003	0.03	0.00, 0.05	0.026
**Household size**	-0.03	-0.04, 0.01	0.002	-0.04	-0.05, -0.02	<0.001	0.00	-0.02, 0.01	0.588
**Mass media exposure**	0.10	0.00, 0.19	0.047	0.09	0.00, 0.17	0.048	0.17	0.10, 0.25	<0.001
**Wave 2 (Ref: Wave 1)**	0.79	0.72, 0.87	<0.001	0.11	0.04, 0.18	0.001	0.55	0.49, 0.60	<0.001
**Number of Observations**	7882			7882			7882		

Note: Robust 95 per cent confidence intervals.

Abbreviations

UDAYA (Understanding Lives of Adolescents and Young Adults)

AGYW (Adolescent Girls and Young Women

Declarations

Funding. This work was not funded.

Competing Interests. *The authors report there are no competing interests to declare.*

Availability of data and materials:

Availability of data and materials: The data that support the findings of this study are openly available in Harvard Dataverse at https://doi.org/10.7910/DVN/RRXQNT (Council, 2018)

Author contributions:

Lakshmi Gopalakrishnan**:** Conceptualization, Methodology, Writing-Original draft preparation, Reviewing and Editing.

Stefano Bertozzi: Supervision, Writing-Reviewing and Editing.

Sophia Rabe-Hesketh**:** Oversight of data analysis and methodology, Supervision, Writing-Reviewing and Editing.

All authors have read and provided feedback on the manuscript

References

1. Field E, Ambrus A. Early Marriage, Age of Menarche, and Female Schooling Attainment in Bangladesh. J Polit Econ. 2008 Oct 1;116(5):881–930.

2. John NA, Edmeades J, Murithi L. Child marriage and psychological well-being in Niger and Ethiopia. BMC Public Health. 2019 Aug 1;19(1):1029.

3. Rao S, Finnoff K. Marriage Migration and Inequality in India, 1983–2008. Popul Dev Rev. 2015;41(3):485–505.

4. Das Gupta M. Life Course Perspectives on Women's Autonomy and Health Outcomes. Am Anthropol. 1995;97(3):481–91.

5. Das Gupta M. Life course perspectives on women's autonomy and health outcomes. Health Transit Rev. 1996;6:213–31.

6. Kandiyoti D. Bargaining with Patriarchy. Gend Soc. 1988 Sep 1;2(3):274–90.

7. Mathur S, Greene M, Malhotra A. Too Young To Wed: The Lives, Rights and Health of Young Married Girls. 2003.

8. Bloom SS, Wypij D, Das Gupta M. Dimensions of women's autonomy and the influence on maternal health care utilization in a north Indian city. Demography. 2001 Feb;38(1):67–78.

9. Jejeebhoy SJ, Halli SS. Marriage Patterns in Rural India: Influence of Sociocultural Context. In: The Changing Transitions to Adulthood in Developing Countries: Selected Studies [Internet]. Washington DC.: The National Academic Press; 2005 [cited 2020 May 17]. Available from: https://www.nap.edu/read/11524/chapter/7

10. International Institute for Population Sciences. National Family Health Survey 4, India 2015-2016 Fact Sheet. Deemed University, Mumbai; 2017.

11. Jejeebhoy SJ, Santhya KG, Zavier AJF. Demand for contraception to delay first pregnancy among young married women in India. Stud Fam Plann. 2014 Jun;45(2):183–201.

12. Pande RP, Falle TY, Rathod S, Edmeades J, Krishnan S, Pande RP, et al. 'If your husband calls, you have to go': understanding sexual agency among young married women in urban South India. Sex Health. 2011 Jan 24;8(1):102–9.

13. Prakash R, Singh A, Pathak PK, Parasuraman S. Early marriage, poor reproductive health status of mother and child well-being in India. BMJ Sex Reprod Health. 2011 Jul 1;37(3):136–45.

14. Singh A, Singh A, Mahapatra B. The consequences of unintended pregnancy for maternal and child health in rural India: evidence from prospective data. Matern Child Health J. 2013 Apr;17(3):493–500.

15. Singh A, Chalasani S, Koenig MA, Mahapatra B. The consequences of unintended births for maternal and child health in India. Popul Stud. 2012 Nov 1;66(3):223–39.

16. Chaaban J, Cunningham W. Measuring the Economic Gain of Investing in Girls: The Girl Effect Dividend [Internet]. The World Bank; 2011 [cited 2022 Jan 31]. 38 p. (Policy Research Working Papers). Available from: https://elibrary.worldbank.org/doi/abs/10.1596/1813-9450-5753

17. Jayachandran S. The Roots of Gender Inequality in Developing Countries. Annu Rev Econ. 2015;7(1):63–88.

18. Mitra A. Son Preference in India: Implications for Gender Development. J Econ Issues. 2014 Dec 1;48(4):1021–37.

19. Nanda P, Gautam A, Verma R, Khanna A, Khan N, Brahme D, et al. Study on Masculinity, Intimate Partner Violence and Son Preference in India. New Delhi, India: International Center for Research on Women (ICRW); 2014.

20. Ebenstein A. Patrilocality and Missing Women. SSRN Electron J [Internet]. 2014 [cited 2020 Dec 31]; Available from: http://www.ssrn.com/abstract=2422090

21. Milazzo A. Why are adult women missing? Son preference and maternal survival in India. J Dev Econ. 2018 Sep 1;134:467–84.

22. Reed MN. Reproductive transitions and women's status in Indian households. Popul Stud. 2021 Sep 2;75(3):325–41.

23. Kabeer N. Resources, Agency, Achievements: Reflections on the Measurement of Women's Empowerment. Dev Change. 1999;30(3):435–64.

24. Narayan D. Empowerment and Poverty Reduction: A Sourcebook [Internet]. Washington DC: The World Bank; 2002 [cited 2022 May 5]. Available from: https://openknowledge.worldbank.org/handle/10986/15239

25. Malhotra A, Schuler SR. Women's empowerment as a variable in international development. In: Measuring empowerment: Cross-disciplinary perspectives. 1(1). 2005. p. 71–88.

26. Upadhyay UD, Gipson JD, Withers M, Lewis S, Ciaraldi EJ, Fraser A, et al. Women's empowerment and fertility: A review of the literature. Soc Sci Med. 2014 Aug 1;115:111–20.

27. Do M, Kurimoto N. Women's empowerment and choice of contraceptive methods in selected African countries. Int Perspect Sex Reprod Health. 2012 Mar;38(1):23–33.

28. Prata N, Fraser A, Huchko MJ, Gipson JD, Withers M, Lewis S, et al. Women's empowerment and family planning: a review of the literature. J Biosoc Sci. 2017 Nov;49(6):713–43.

29. Pratley P. Associations between quantitative measures of women's empowerment and access to care and health status for mothers and their children: A systematic review of evidence from the developing world. Soc Sci Med. 2016 Nov 1;169:119–31.

30. Santhya KG, Acharya R, Pandey N, Singh S, Rampal S, Zavier AJ, et al. Understanding the lives of adolescents and young adults (UDAYA) in Bihar, India. Poverty Gend Youth

[Internet]. 2017 Jan 1; Available from: https://knowledgecommons.popcouncil.org/departments_sbsr-pgy/544

31. Kristman V, Manno M, Côté P. Loss to follow-up in cohort studies: How much is too much? Eur J Epidemiol. 2004;19(8):751–60.

32. Council P. UDAYA, Adolescent Survey, Bihar and Uttar Pradesh, 2015-16 [Internet]. Harvard Dataverse; 2018 [cited 2022 Oct 18]. Available from: https://dataverse.harvard.edu/dataset.xhtml?persistentId=doi:10.7910/DVN/RRXQNT

33. Agarwal B. ”Bargaining” and Gender Relations: Within and Beyond the Household. Fem Econ. 1997 Jan 1;3(1):1–51.

34. Jensen R, Oster E. The Power of TV: Cable Television and Women's Status in India [Internet]. National Bureau of Economic Research; 2007 Aug [cited 2021 Jan 1]. Report No.: w13305. Available from: https://www.nber.org/papers/w13305

35. Jejeebhoy SJ, Sathar ZA. Women's Autonomy in India and Pakistan: The Influence of Religion and Region. Popul Dev Rev. 2001;27(4):687–712.

36. Allendorf K. Going nuclear? Family structure and young women's health in India, 1992-2006. Demography. 2013 Jun;50(3):853–80.

37. Anukriti S, Herrera-Almanza C, Pathak PK, Karra M. Curse of the Mummy-ji: The Influence of Mothers-in-Law on Women in India†. Am J Agric Econ. 2020;102(5):1328–51.

38. Desai S, Kiersten J. Women's decision making and child health: Familal and social hierachies [Internet]. 2005 [cited 2022 Feb 16]. Available from: https://mpra.ub.uni-muenchen.de/111171/

39. Stata Statistical Software: Release 15. College Station, TX: StataCorp LLC; 2017.

40. Dhanaraj S, Mahambare V. Family structure, education and women's employment in rural India. World Dev. 2019 Mar 1;115:17–29.

41. Roy TK, Niranjan S. Indicators of women's empowerment in India. Asia-Pac Popul J. 2005 Nov 18;19(3):23–38.

42. Malhotra A, Vanneman R, Kishor S. Fertility, Dimensions of Patriarchy, and Development in India. Popul Dev Rev. 1995;21(2):281–305.

43. Harris-Fry H, Shrestha N, Costello A, Saville NM. Determinants of intra-household food allocation between adults in South Asia – a systematic review. Int J Equity Health. 2017 Jun 21;16(1):107.

44. MacQuarrie K. Women's Empowerment and Family Formation across the Life Course in Madhya Pradesh, India: The Influence of Time-varying and Fixed Empowerment Resources. In Marrakech, Morocco; 2009. Available from: https://iussp2009.princeton.edu/papers/91879

45. Dixit A, Bhan N, Benmarhnia T, Reed E, Kiene SM, Silverman J, et al. The association between early in marriage fertility pressure from in-laws' and family planning behaviors, among married adolescent girls in Bihar and Uttar Pradesh, India. Reprod Health. 2021 Mar 9;18(1):60.

46. Brunson J. Confronting maternal mortality, controlling birth in Nepal: The gendered politics of receiving biomedical care at birth. Soc Sci Med. 2010 Nov 1;71(10):1719–27.

47. Samari G. First birth and the trajectory of women's empowerment in Egypt. BMC Pregnancy Childbirth. 2017 Nov 8;17(2):362.

48. Lim SS, Dandona L, Hoisington JA, James SL, Hogan MC, Gakidou E. India's Janani Suraksha Yojana, a conditional cash transfer programme to increase births in health facilities: an impact evaluation. The Lancet. 2010 Jun 5;375(9730):2009–23.

49. Kishore A, Spears D. Having a Son Promotes Clean Cooking Fuel Use in Urban India: Women's Status and Son Preference. Econ Dev Cult Change [Internet]. 2015 Jul 17 [cited 2022 Feb 7]; Available from: https://www.journals.uchicago.edu/doi/abs/10.1086/676330

50. Gupta R, Nimesh R, Singal GL, Bhalla P, Prinja S. Effectiveness of India's National Programme to save the girl child: experience of Beti Bachao Beti Padao (B3P) programme from Haryana State. Health Policy Plan. 2018 Sep 1;33(7):870–6.

51. Ministry of Health & Family Welfare, Government of India. National Health Mission [Internet]. 2016 [cited 2022 Mar 6] p. 11–23. Available from: https://main.mohfw.gov.in/sites/default/files/56987532145632566578.pdf

52. Narayan-Parker D. Measuring Empowerment: Cross-disciplinary Perspectives. World Bank Publications; 2005. 501 p.

53. Richardson RA. Measuring Women's Empowerment: A Critical Review of Current Practices and Recommendations for Researchers. Soc Indic Res. 2018 Jun 1;137(2):539–57.

54. Ghuman SJ, Lee HJ, Smith HL. Measurement of women's autonomy according to women and their husbands: Results from five Asian countries. Soc Sci Res. 2006 Mar 1;35(1):1–28.

55. UN Women, editor. Turning promises into action: gender equality in the 2030 Agenda for Sustainable Development. New York, NY: UN Women; 2018. 337 p.

Figure

Click here to access/download;Figure;Figure1.docx

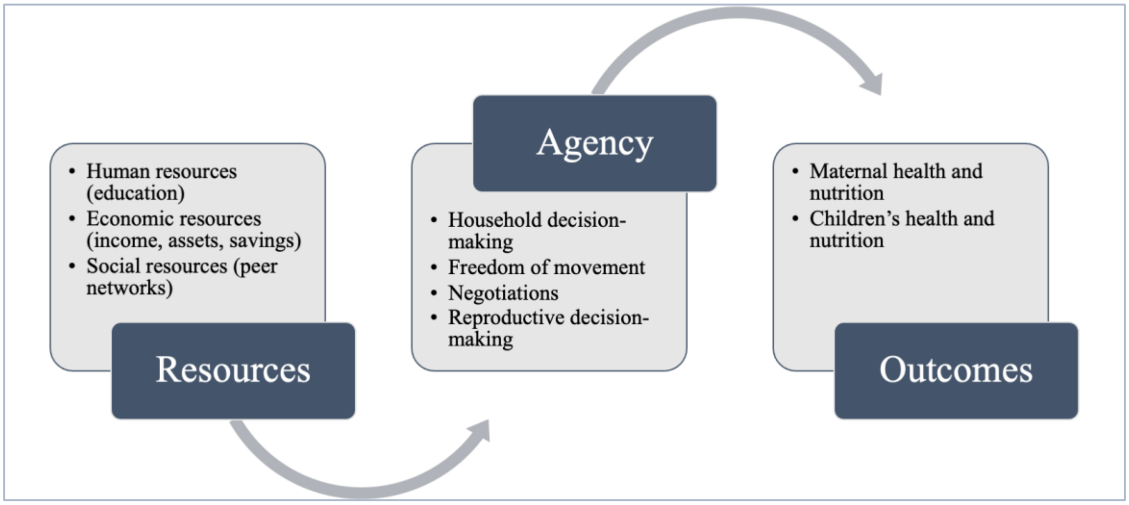

Figure 1: Women's empowerment conceptualized using Kabeer's framework

Click here to access/download
Supporting Information
STROBE-checklist-v4-combined-PlosMedicine.docx

Click here to access/download
Supporting Information
PLOSOne_Clinical_Studies_Checklist.docx
