# Supplementary material for: Role of marriage, motherhood, son preference on adolescent girls’ and young women’s empowerment: Evidence from a panel study in India
Source: PLoS One. 2023 Sep 28;18(9):e0292084. doi: 10.1371/journal.pone.0292084 (PMC10538655; doi:10.1371/journal.pone.0292084)
Supplement: Attachment — Submitted filename: renamed_7aacb.docx [file pone.0292084.s005.docx]

# Summary Review: Role of marriage, motherhood, son preference on adolescent girls’ empowerment: Evidence from a panel study in India

| 1. Importance of article/Relevance and Appeal to national / international scholarly | Excellent | **Good** | Moderate | Poor |  |
| --- | --- | --- | --- | --- | --- |
| 2. Title of the study | Excellent | **Good** | Moderate | Poor |  |
| 3. Original and Independent Research | Excellent | **Good** | Moderate | Poor |  |
| 4. Presentation and readability | Excellent | **Good** | Moderate | Poor |  |
| 5. Statement of problem(s)/aim(s)/objective(s) | Excellent | Good | Moderate | Poor | **Not Available** |
| 6. Literature review | Excellent | Good | Moderate | Poor | **Not Available** |
| 7. Appropriateness of (if applicable)    7.1. Research plan and design      7.2. Data presentation/Discussion 7.3. Conclusion/Recommendations |  | | | |  |
|  | Excellent | **Good** | Moderate | Poor |  |
|  | Excellent | **Good** | Moderate | Poor |  |
|  | Excellent  Excellent | **Good**  **Good** | Moderate  Moderate | Poor  Poor |  |
| 8. To what extent is the line of argumentation in the article clear, cohesive and logical? | Excellent | **Good** | Moderate | Poor |  |
| 9. Contribution to theory | Excellent | Good | Moderate | Poor |  |
| 10. Contribution to practice | Excellent | Good | Moderate | Poor |  |

**NOTE:**

- This paper will be better situated if it follows the chronological order of writing an Introduction.
