# Supplementary material for: Role of marriage, motherhood, son preference on adolescent girls’ and young women’s empowerment: Evidence from a panel study in India
Source: PLoS One. 2023 Sep 28;18(9):e0292084. doi: 10.1371/journal.pone.0292084 (PMC10538655; doi:10.1371/journal.pone.0292084)
Supplement: Attachment — Submitted filename: Response to Reviewers_August26.docx [file pone.0292084.s007.docx]

We thank the Editor for his careful review and comments. Please find below our responses. Thank you.

- You may not need to state hypotheses based on research questions, since you did not present your results based on the stated hypotheses. Rather, you can just state one single hypothesis that captures the aim of the study

I deleted the hypotheses and summarized it in a single sentence as recommended by the Editor.

- Revision of the title is not sufficient...there is the need to pay close attention to other issues to ensure a smooth flow from the title throughout the manuscript. For example, there is the need to give clearer explanation as regarding the use of the 2 waves of data. Let it be clear that it is only the adolescent girls you considered in the first wave, while you considered both adolescents and young women in the second wave, due to age progression from the first to the second wave.
  - I have revised the title a little bit further. Also, throughout the paper, where reference to Wave 1 is being discussed, I changed it to adolescent girls. And for Wave 2 related discussion, I use AGYW – to encompass the age ranges of both adolescent girls and young women (AGYW).
- There is the need to further revise the Methods and Analysis sections to remove unnecessary repetitions, that make the methods section clumsy to understand.
  - We are sorry the methods section was verbose making it hard to read. I have now amended the methods and analysis section to make it concise. I moved the estimating equations to Supplementary Material. Please refer to pages 17-19 for edits.
